# Leaf-vein-inspired multi-organ microfluidic chip for modeling breast cancer CTC organotropism

**DOI:** 10.3389/fonc.2025.1602225

**Published:** 2025-05-29

**Authors:** Liuyin Liu, Xiaoli Qu, Zhe Wang, Cheng Ji, Rui Ling, Changjiao Yan

**Affiliations:** ^1^ Department of Thyroid, Breast, and Vascular Surgery, Xijing Hospital, The Fourth Military Medical University, Xi’an, Shaanxi, China; ^2^ State Key Laboratory for Manufacturing Systems Engineering, Xi’an Jiaotong University, Xi’an, China

**Keywords:** breast cancer, circulating tumor cell, leaf vein architecture, multi-organ microfluidic chip, organotropism

## Abstract

**Objective:**

Breast cancer is characterized by a high tendency for organ-specific metastasis. This study aims to develop a multi-organ metastasis model for circulating tumor cells (CTCs) of breast cancer to explore their organotropism in common target organs, including the liver, bone, and lung.

**Methods:**

We fabricated a biomimetic microfluidic organ-on-a-chip inspired by leaf veins. In this system, three-dimensional cultures of human hepatocyte LO2 cells, human bone marrow-derived mesenchymal stem cells, and human fetal lung fibroblast 1 cells were established in separate chambers to mimic liver, bone, and lung microenvironments, respectively. Then, various breast cancer subtypes (MCF-7, SKBR3, MDA-MB-231) were perfused through the system. We quantified their invasive cell numbers and organ-specific localization in each organ. Further, MDA-MB-231 cells overexpressing metastasis-related genes (CXCR4, claudin-2, Linc-ZNF469-3) were tested. Additionally, the integration of tumor organoids with microfluidic chips was employed to evaluate the predictive capacity of this model for patient-specific metastatic patterns.

**Results:**

There are significant differences in the number of invasive cells and organ-specific localization among different breast cancer subtypes in each organ. MCF-7 cells show the highest invasion and most prominent localization in bone; SKBR3 cells in liver and lung. MDA-MB-231 cells have no obvious difference in organotropism among the three organs, but their invasive numbers are higher than those of MCF-7 cells. CXCR4-OE, claudin-2-OE, and Linc-ZNF469-3-OE MDA-MB-231 cells demonstrate the highest invasion and most prominent localization in bone, liver, and lung respectively. Organoid cells derived from a breast cancer patient with pulmonary metastasis at initial diagnosis, when perfused into the system, selectively invaded the lung organ, but did not invade the liver, bone, or control pores.

**Conclusion:**

This leaf-vein-inspired multi-organ microfluidic chip demonstrates significant application value for studying breast cancer CTC organotropism and serves as a powerful predictive tool for early warning of high-risk organ metastasis.

## 1 Introduction

According to the latest Global Cancer Statistics 2024 report, breast cancer remains the most prevalent malignancy among women worldwide and the leading cause of cancer-related deaths in females (1). Distant organ metastasis is the primary contributor to mortality in breast cancer patients (2). Circulating tumor cells (CTCs), acting as “seed cells” for cancer metastasis, detach from primary tumors, invade the vascular system, and subsequently colonize target organ microenvironments to form secondary tumors, driving the entire metastatic cascade (3). However, conventional research methodologies fail to adequately simulate the dynamic behaviors of CTCs during multi-organ-specific adhesion: two-dimensional cultures lack cell-cell and cell-matrix interactions, limiting their ability to replicate physiological and pathological processes *in vivo* (4); animal models suffer from interspecies variability and lack real-time visualization capabilities (5). Thus, developing an *in vitro* platform enabling real-time observation of CTC organotropism is critical for elucidating metastatic mechanisms and advancing precision therapeutic strategies.

Recent advancements in microfluidic technology have provided novel platforms for metastasis research (6). Compared to traditional approaches, microfluidic systems offer advantages including short experimental duration, minimal sample consumption, and cost-effectiveness. Importantly, they enable precise control of fluidic microenvironments to mimic physiological conditions, such as nutrient delivery, waste removal, and intercellular interactions, thereby establishing three-dimensional (3D) culture environments that better approximate *in vivo* states (7). S. Bersini et al. developed a 3D microfluidic model to simulate breast cancer bone metastasis (8). By culturing osteocytes in a 3D matrix, this study reconstructed bone-specific tumor microenvironments and effectively modeled breast cancer cell migration toward bone tissues, providing a reliable *in vitro* platform for investigating bone metastasis mechanisms and identifying therapeutic targets. However, this work focused solely on single-organ (bone) metastasis and failed to recapitulate the selective metastasis process among multiple organs. Furthermore, the microfluidic channel design lacked sufficient biomimicry to replicate intricate vascular architectures.

Inspired by the shared fluid transport principles (Murray’s law) between plant leaf venation and mammalian vascular systems, recent studies have leveraged biomimetic leaf vein networks to address these limitations. Jiankang He et al. developed perfusable microfluidic chips by precisely replicating natural leaf venation patterns onto silicon substrates through microreplication techniques (9). These chips were applied to construct efficient vascularized transport systems for high-throughput cellular experiments (10). Subsequent work by this team demonstrated the chip’s excellent defect-resistant fluid transport capability. They determined the optimal perfusion parameters for forming an endothelialized capillary network *in vitro* and revealed a strong correlation between cancer cell adhesion and blood flow-induced shear stress (11). These studies demonstrated that the leaf-vein-inspired network provides a more physiologically relevant platform compared to conventional microfluidic designs, particularly in terms of fluid dynamics and nutrient distribution. This team further designed a leaf vein-inspired chip with organ chambers to mimic human vascular systems interconnected with vascularized organs, validating its utility in metastasis research using pancreatic cancer cells that metastasized to bone and liver tissues (12). Nevertheless, this study primarily focused on vascular-organ connectivity rather than investigating pancreatic cancer cell organotropism, leaving gaps in modeling the “homing-colonization” phase of multi-organ-specific adhesion.

**Figure 1:**
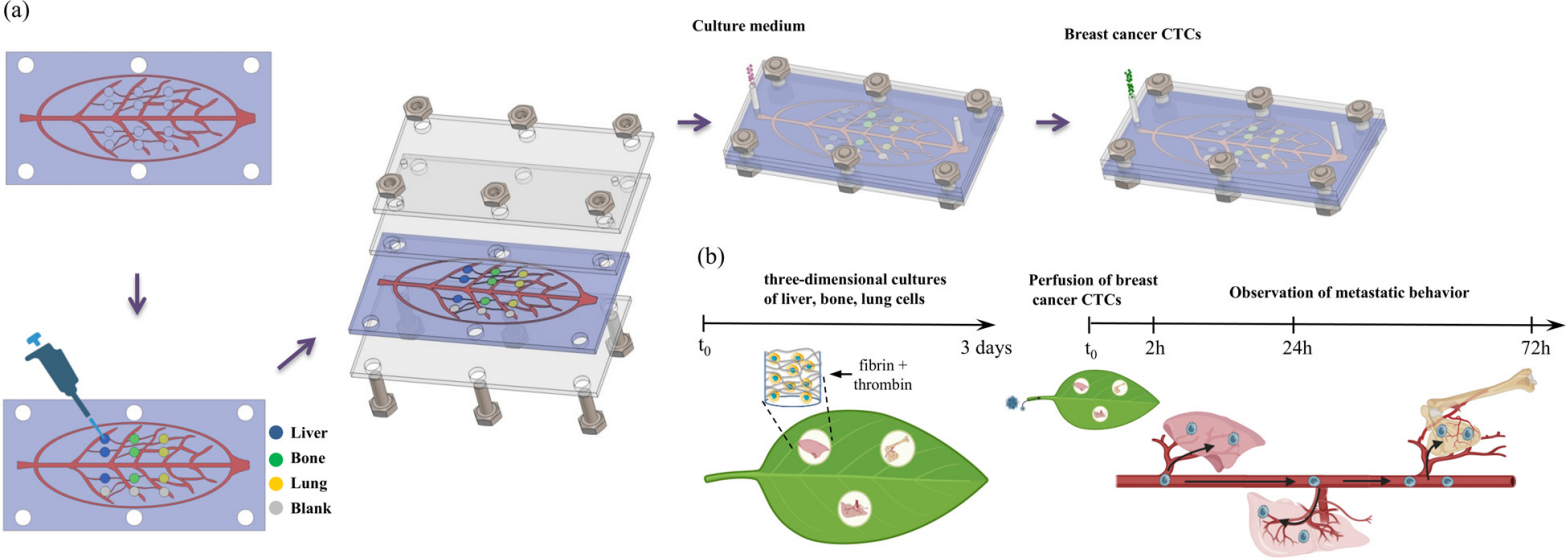
**(a)** Schematic illustration of the assembly of the leaf-venation microfluidic chip. **(b)** Schematic diagram of the biomimetic microfluidic chip to study organ-specific metastasis of breast cancer CTCs.

Building upon the prior research of the leaf-vein microfluidic chip’s hydraulics and biomimetic properties, and noting breast cancer’s unique molecular traits and organotropism, we collaborated with Prof. Jiankang He’s team to develop a leaf vein-inspired multi-organ chip model. As shown in **Figure 1**, it explores the organotropism of different breast cancer subtypes in common metastatic organs like the liver, bone, and lung. The model’s validity was further verified using breast cancer cells overexpressing metastasis-specific genes (CXCR4, claudin-2, Linc-ZNF469-3) and a patient-derived organoid (PDO) from a patient with advanced metastatic disease. This platform establishes a novel paradigm for deciphering CTC organotropism mechanisms.

## 2 Materials and methods

### 2.1 Cells and reagents

MDA-MB-231, MCF-7, and SKBR3 cells were purchased from the Chinese Academy of Sciences Cell Bank (Shanghai, China). GFP+ MDA-MB-231 breast cancer cells were kindly provided by the State Key Laboratory of Cancer Biology, Department of Biochemistry and Molecular Biology, Fourth Military Medical University (Xi’an, China). These cells were cultured in Dulbecco’s Modified Eagle’s Medium (DMEM, Corning, USA) supplemented with 10% (v/v) fetal bovine serum (FBS, Newzerum, New Zealand) and 1% (v/v) penicillin-streptomycin (Hyclone, USA). LO2 cells (Chinese Academy of Sciences Cell Bank, Shanghai, China) were cultured in RPMI 1640 (Corning, USA) supplemented with 10% (v/v) FBS and 1% (v/v) penicillin-streptomycin. This cell line was originally derived from normal human liver tissue. HFL-1 (Human Fetal Lung Fibroblast 1) cells were obtained from the Cell Bank of the Chinese Academy of Sciences and cultured in F-12K medium (Sai Bai Kang Biotechnology Co., Ltd., Shanghai, China) supplemented with 10% FBS and 1% penicillin/streptomycin. HFL-1 cells were used at passages 2 through 7. Human Bone Marrow-Derived Mesenchymal Stem Cells (h-MSCs, Cell Bank of the Chinese Academy of Sciences, Shanghai, China) were cultured in serum-free primary mesenchymal stem cell medium (Sai Bai Kang Biotechnology Co., Ltd., Shanghai, China). h-MSCs were used at passages 2 through 7. Patient-derived organoids (PDOs) were established from specimens obtained from a breast cancer patient with pulmonary metastasis at initial diagnosis (age 42). The patient gave informed consent and the study was approved by the Ethical Committee of Xijing Hospital (KY20252024-F-1).

### 2.2 Construction of overexpressing MDA-MB-231 cells

Cell seeding: MDA-MB-231 cells were suspended in complete DMEM medium and seeded into 6-well plates at a density of 2×10^5^ cells per well. Cells were cultured at 37°C for 16–24 h until reaching 20–30% confluence. Viral infection: Lentiviruses overexpressing CXCR4, claudin-2, and Linc-ZNF469–3 were purchased from Shanghai GeneChem Co., Ltd. (China). The multiplicity of infection (MOI) for MDA-MB-231 cells was 10. The viral stock was diluted 10-fold, and the required volume was calculated using the formula: Viral volume = (MOI × cell number)/viral titer. After 12–16 h of incubation at 37°C, the medium was replaced with complete DMEM. At 48 h post-infection, cells with fluorescence were observed under a microscope, and puromycin (2 μg/mL) was added to select successfully infected cells. After 48–72 h of puromycin selection, cells with fluorescence exceeding 90% were subjected to qRT-PCR validation.

### 2.3 Quantitative RT-PCR

Total RNA was extracted using TRIzol reagent (TaKaRa, Japan), and cDNA synthesis was performed using a reverse transcription kit (Yeasen Biotechnology Co., Ltd., Shanghai, China). Briefly, 2,000 ng of RNA was reverse transcribed into cDNA in a 20 μL reaction volume following the manufacturer’s protocol. Real-time quantitative PCR (qPCR) was performed using SYBR Green PCR master mix (Yeasen Biotechnology Co., Ltd., China) on an ABI real-time PCR instrument (Thermo Fisher, USA). β-actin was used as an internal control for normalization. Each sample was run in triplicate to ensure reliability and accuracy. Primer sequences were as follows: CXCR4: F: ATCATCTTCTTAACTGGCATTGTG, R: GCTGTAGAGGTTGACTGTGTAG; Claudin-2: F: GGCATTATTTCTTCCCTGTTC, R: TTGGTAGGCATCGTAGTAGTTG; Linc-ZNF469-3: F: CAGTCTCTCTGAGGAGCACG, R: GGCCTCAGTTTGTTCAGGAGA.

### 2.4 Fabrication of the leaf-venation-inspired microfluidic device

The leaf-venation-inspired microfluidic device was fabricated using standard soft lithography as previously described (13). Briefly, a photomask of the leaf-venation network was manufactured based on a digital model. HMDS was spin-coated onto a silicon wafer at 1,000 rpm for 10 s and cured at 95°C for 15 min. A photoresist (EPG533, Everlight Chemical Industrial Co., Taiwan) was then spin-coated at 1,500 rpm for 40 s. After curing, the coated wafer was exposed to UV light for 6 s, and the exposed regions were dissolved in 0.5% NaOH. Dry etching was performed using an ICP etcher (Oxford, ICP180) to achieve a vein depth of 300 µm and a chamber depth of 600 µm (chamber diameter: 2 mm). The wafer was treated with octafluorocyclobutane (C4F8) to facilitate demolding. A mixture of Sylgard 184 silicone elastomer (10:1 weight ratio) was degassed, poured onto the wafer, and cured at 60°C for 4 h. The PDMS (Polydimethylsiloxane) layer with the negative pattern of the leaf-venation microchannel was peeled off, trimmed, and coated with C4F8. This PDMS layer served as a mold to produce the final microchannel substrate with chambers.

### 2.5 Assembly of the multi-organ leaf-venation microfluidic chip

Holes were punched in the PDMS layers using a 4 mm puncher, aligning with the screw holes on the polymethyl methacrylate (PMMA) layer. The PDMS layers were sterilized using an autoclave. After drying, 4 μL of fibrin gel containing cell suspensions (final concentration: fibrin (Millipore, USA) 3 mg/mL, thrombin (Solarbio, China) 1.5 U/mL; red fluorescent fibrin: normal fibrin=1:20; aprotinin (Proteintech Group, Inc., China) 0.15 U/mL) was added to the chambers. The gel was crosslinked at 37°C for 10 min. Cell densities were as follows: LO2 cells at 0.9×10^6^ cells/mL, h-MSCs and HFL-1 cells at 0.8×10^6^ cells/mL, and a blank group without cells. After gelation, the PDMS layers were assembled using a sandwich method with PMMA clamps. The device was connected to a syringe via a 23G needle and PTFE tubing (inner diameter: 0.56 mm). A 1:1:1 mixture of LO2, h-MSC, and HFL-1 culture media was perfused at 0.2 mL/h.

### 2.6 Perfusion of breast cancer CTCs into the multi-organ microfluidic chip

Seven types of breast cancer cells were studied for their metastatic tropism: MCF-7, SKBR3, MDA-MB-231, CXCR4 OE 231, claudin-2 OE 231, Linc-ZNF469–3 OE 231, and PDOs. MCF-7 and SKBR3 were stained with Cell-Tracker Green CMFDA (Maokang Biotechnology Co., Ltd., Shanghai, China) at 10 µM for 30–45 min. After staining, cells were incubated in complete medium for 4 h before perfusion.

Breast cancer cells were resuspended in complete medium at 1.4×10^7^ cells/mL and loaded into a 1 mL syringe. The syringe was connected to the microfluidic system, and 200 µL of cell suspension was slowly injected under an inverted microscope. The device was incubated at 37°C for 6 h, followed by perfusion at 0.2 mL/h using a 1:1:1:1 mixture of LO2, h-MSC, HFL-1, and breast cancer cell culture media. Organotropism behavior was observed at 2, 24, and 72 h using confocal microscopy.

### 2.7 Statistical analysis

Effective chambers were defined as those with ≥5 breast cancer cells within 100 µm of the chamber gel after 2 or 24 h of perfusion. Image J was used to count migrated cells and measure the maximum localization extent. Statistical analysis was performed using SPSS 26.0. Multivariate ANOVA was used for group comparisons, with p<0.05 considered statistically significant.

## 3 Results

### 3.1 Construction of a leaf-vein-inspired multi-organ microfluidic chip with liver, bone and lung

**Figure 2:**
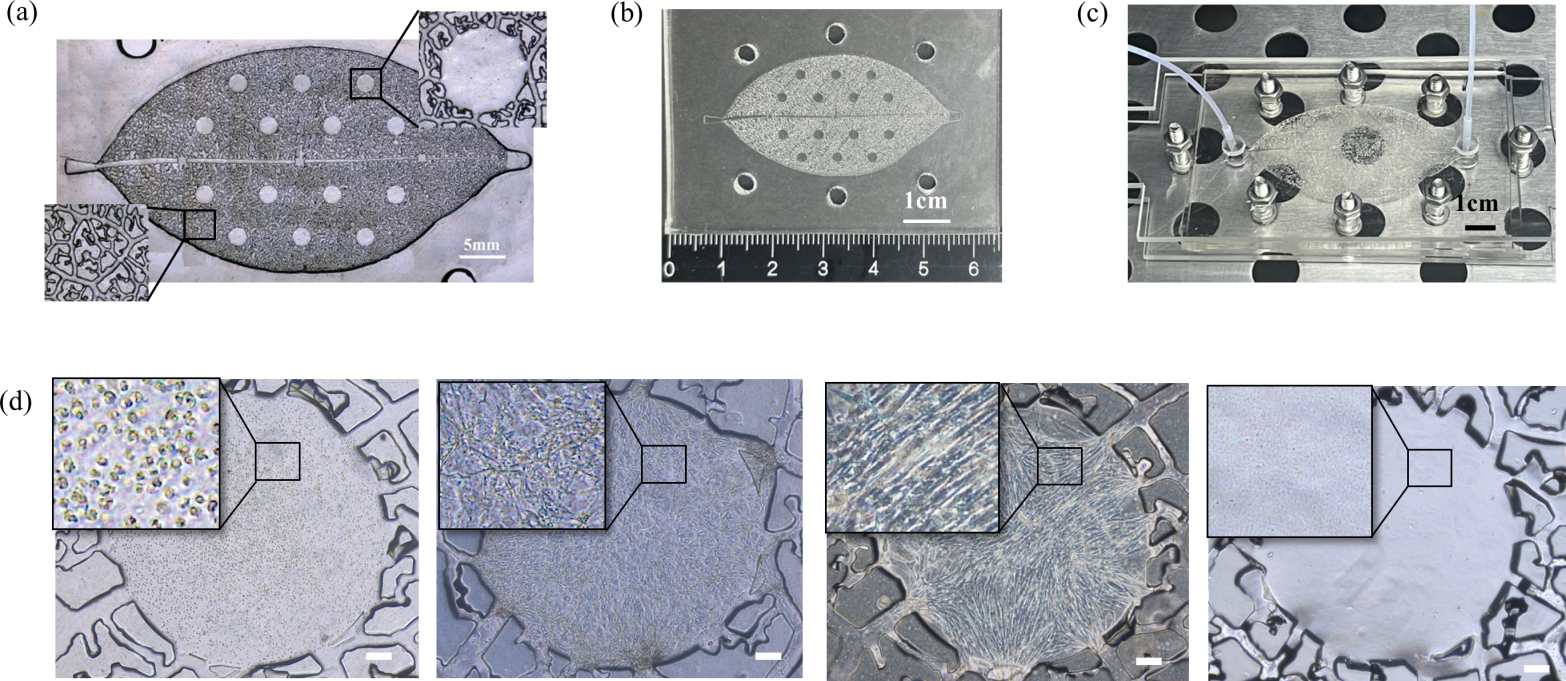
**(a)** Microscope image of the leaf-venation framework. **(b)** Photograph of the PDMS-fabricated leaf-venation framework. **(c)** Photograph of the leaf-venation microfluidic chip system under perfusion in an incubator. **(d)** Bright field images of cells in the microfluidic chip chamber after 3 days of perfusion. The cells from left to right are respectively: LO2 cells (Human normal liver cells), h-MSCs (Human Bone Marrow-Derived Mesenchymal Stem Cells), HFL-1 (Human Fetal Lung Fibroblast 1) cells, Blank gel without cells. Scale bar: 200 μm.

Based on the structural similarity between plant vascular systems and mammalian circulatory systems in bionics, we developed a unidirectional high-throughput bionic leaf-vein microfluidic chip to build a multi-organ co-culture model and explore breast cancer metastasis tendencies. The fabrication process of the chip is illustrated in **Figure 1a**. First, a leaf-venation-like channel structure (channel depth: 300 µm) was created on a silicon template using standard photolithography. The branching network included randomly distributed chambers (diameter: 2 mm, depth: 600 µm), which were connected to the secondary structures of the leaf-venation microchannels to ensure communication between the chambers and the microchannels (**Figures 2a, b**). A PDMS replica molding process was used to produce the base layer with recessed microstructures. Fibrin gels containing human cells were then embedded in the chambers: the experimental groups included LO2, h-MSCs, and HFL-1 cells to simulate 3D liver, bone, and lung organ microenvironments, respectively; the control group consisted of cell-free fibrin gels. After the fibrin gel was crosslinked with thrombin to form a hydrogel, the chip was integrated using a lamination encapsulation process: the leaf-vein PDMS layer and a flat PDMS cover layer were pressed together using PMMA clamps to form a closed microchannel system. A dynamic perfusion system (flow rate: 0.2 mL/h) was established by connecting the biomimetic leaf-venation microfluidic chip to an external pump via PTFE tubing (**Figure 2c**). Morphological observations after 3 days of pre-culture (**Figure 2d**) showed that LO2, h-MSCs, and HFL-1 cells were uniformly distributed within the gel and exhibited well-extended morphologies, indicating the system’s ability to maintain a stable *in vitro* microenvironment. Three days after seeding cells into the chamber, circulating breast cancer cells were perfused into the leaf-venation inlet, and their tropism toward liver, bone, lung, and blank chambers was observed at 2, 24, and 72 h post-perfusion.

### 3.2 Organotropism of different breast cancer CTC subtypes to liver, bone, and lung

**Figure 3:**
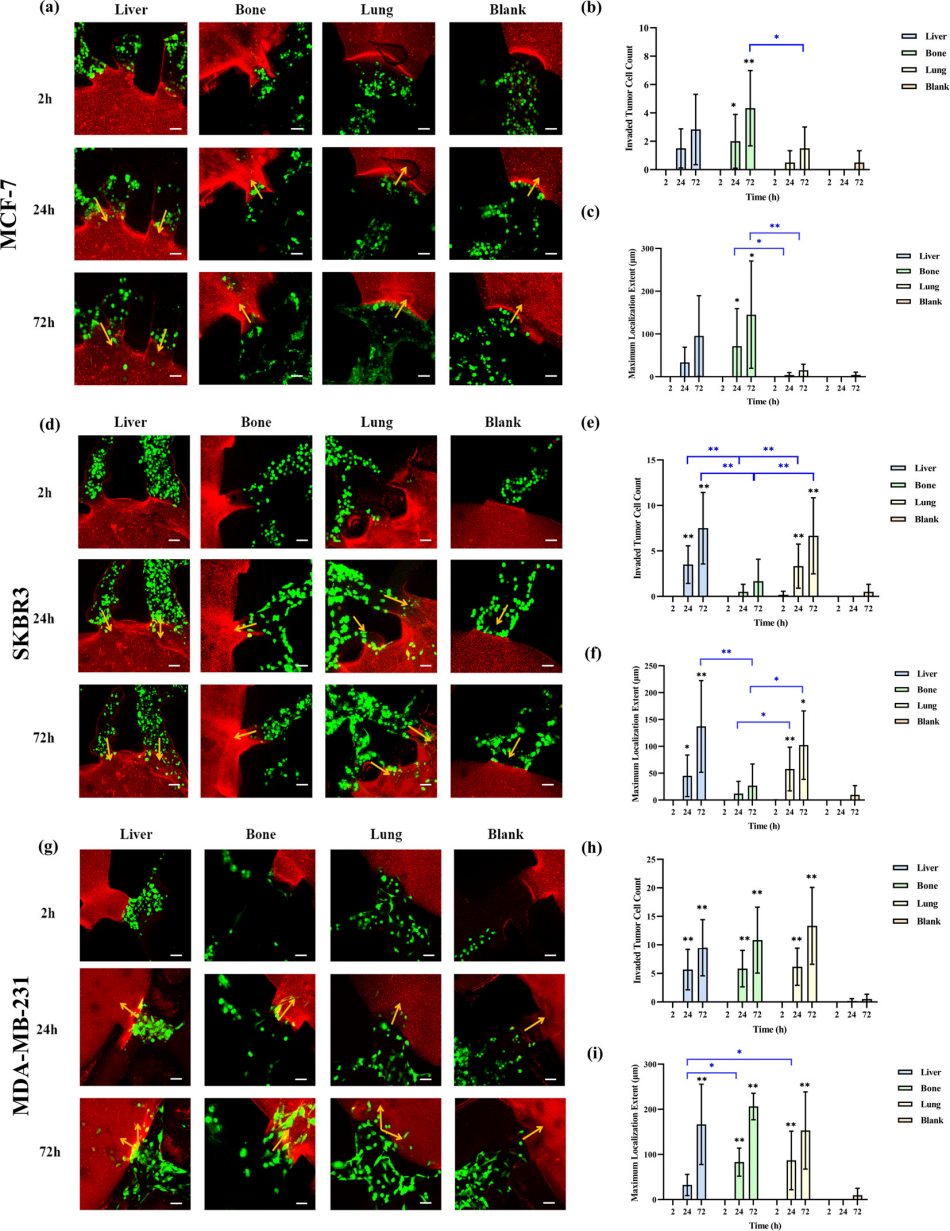
Comparison of the organotropism of different breast cancer subtypes to liver, bone and lung. **(a)** Confocal laser microscopy of chambers and adjacent channels at 2, 24 and 72 h after MCF-7 perfusion; **(b)** Number of MCF-7 cells invading into liver, bone, lung and blank chambers; **(c)** Maximum localization extent of MCF-7 cells into liver, bone, lung and blank chambers; **(d)** Confocal laser microscopy of chambers and adjacent channels at 2, 24 and 72 h after SKBR3 perfusion; **(e)** Number of SKBR3 cells invading into liver, bone, lung and blank chambers; **(f)** Maximum localization extent of SKBR3 cells migrating into liver, bone, lung and blank chambers; **(g)** Confocal laser microscopy of chambers and adjacent channels at 2, 24 and 72 h after MDA-MB-231 perfusion; **(h)** Number of MDA-MB-231 cells invading into liver, bone, lung and blank chambers; **(i)** Maximum localization extent of MDA-MB-231 cells migrating into liver, bone, lung and blank chambers. Scale bar: 50 μm. Migration numbers and extents were counted for breast cancer cells entering chambers in individual secondary channels (n=6). In the bar charts for migration numbers and extents, statistical values on individual bars indicate comparisons between breast cancer cell migration numbers or localization extents into organs and those in blank groups at the same time point. Statistical values with lines indicate intergroup comparisons of breast cancer cell migration numbers or extents into organs at the same time point. *p<0.05, **p<0.01.

We investigated the organotropism of three breast cancer subtypes—MCF-7 (hormone receptor-positive), SKBR3 (HER2+), and MDA-MB-231 (triple-negative)—toward liver, bone, and lung using the leaf-venation microfluidic organ-on-a-chip. First, we compared the number of invading cells and the organ-specific localization for each subtype in different organs. As shown in **Figures 3a–c**, 24 hours after perfusion, MCF-7 cells invaded the liver, bone, and lung chambers, but not the blank chambers. Compared to the blank chambers, the number of MCF-7 cells invading the bone chamber was significantly higher (p<0.05), followed by the liver and lung chambers. MCF-7 cells also showed the most prominent localization in the bone chamber, showing statistically significant differences compared to the lung chamber and blank chambers (p <0.05). At 72 hours post-perfusion, the number of invading MCF-7 cells increased in all chambers, including the blank chambers. The bone chamber still exhibited the highest number of invading MCF-7 cells and the most prominent localization, with significant differences compared to the lung and blank chambers (p< 0.05).

As shown in **Figures 3d–f**, SKBR3 cells invaded the liver, bone, and lung chambers 24 hours after perfusion. By 72 hours, the number of invading SKBR3 cells increased in all chambers, including the blank chambers. At both 24 and 72 hours, the number of SKBR3 cells invading the liver and lung chambers was significantly higher than in the blank and bone chambers (p<0.05), and the extent of their localization within these chambers was also greater (p <0.05).

**Figures 3g–i** show that MDA-MB-231 cells invaded the liver, bone, lung, and blank chambers 24 hours after perfusion. Compared to the blank chambers, the number of MDA-MB-231 cells invading the liver, bone, and lung chambers was significantly higher (p<0.05), but there was no difference in the number of cells invading these three organs. In terms of localization extent, MDA-MB-231 cells demonstrated significantly greater localization in the bone and lung chambers compared to the blank and liver chambers (p<0.05). At 72 hours post-perfusion, the number of MDA-MB-231 cells increased in all chambers, and the extent of their localization also increased. Compared to the blank chambers, the number of invading cells and the localization extent were significantly higher in the liver, bone, and lung chambers (p<0.05), but there was no difference among these three organs.

Next, we compared the differences in the number of invading cells and the maximum localization extent among the three subtypes within the same organ. As shown in **Supplementary Figure S1**, 24 and 72 hours after perfusion, the number of MDA-MB-231 cells invading the liver chamber was higher than that of MCF-7 cells (p<0.05), but there was no difference in the maximum localization extent among the three subtypes in the liver chamber. At 24 hours, the number of MDA-MB-231 cells invading the bone chamber was significantly higher than that of MCF-7 and SKBR3 cells (p< 0.01), and the localization extent of MDA-MB-231 cells was significantly greater than that of SKBR3 cells (p<0.05). At 72 hours, the number of MDA-MB-231 cells invading the bone chamber remained higher than that of MCF-7 and SKBR3 cells (p<0.05), and the localization extent of MDA-MB-231 and MCF-7 cells was significantly greater than that of SKBR3 cells (p < 0.05).

At 24 hours post-perfusion, the number of MDA-MB-231 cells invading the lung chamber and their localization extent were significantly greater than those of MCF-7 cells, while there was no difference between SKBR3 cells and the other two subtypes. At 72 hours, the number of MDA-MB-231 cells invading the lung chamber was higher than that of MCF-7 and SKBR3 cells (p<0.05), and the localization extent of MCF-7 cells was significantly shorter than that of SKBR3 and MDA-MB-231 cells (p<0.05). There was no difference in the number of invading cells or the migration distance among the three subtypes in the blank chambers at 24 or 72 hours.

To investigate whether the organotropism behavior of breast cancer cells were influenced by gel structural changes induced by liver, bone, and lung cells, we compared the fibrin gel structures between the experimental and blank groups. Confocal microscopy images revealed no significant differences in gel structure (**Supplementary Figure S2**), suggesting that gel remodeling is not a key factor influencing breast cancer cell localization.

### 3.3 Organotropism of MDA-MB-231 cells overexpressing metastasis-related genes to liver, bone and lung

Breast cancer metastasis to distant organs is regulated by molecular mechanisms. Past studies show that high claudin-2 expression in breast cancer cells increases liver adhesion (14). bone-derived CXCL12 preferentially recruits breast cancer cells with high CXCR4 expression to bone metastasis sites (15). And high Linc-ZNF469–3 expression in triple-negative breast cancer (TNBC) cells promotes lung metastasis (16). Thus, we constructed three overexpressed MDA-MB- 231 cell lines: CXCR4 OE 231, claudin-2 OE 231, and Linc-ZNF469–3 OE 231. q-PCR confirmed successful construction (**Supplementary Figure S3**), and their organotropism to liver, bone and lung on the leaf-vein microfluidic organ chip were studied.

**Figure 4:**
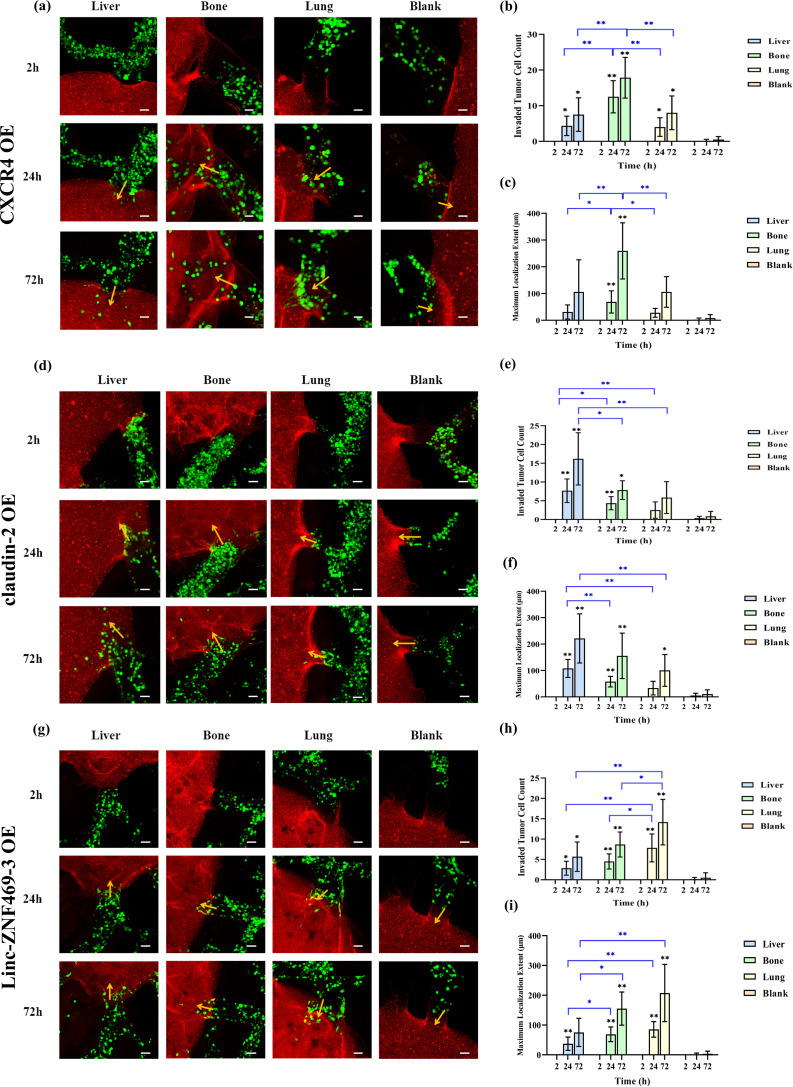
Comparison of the organotropism of different overexpressed MDA-MB-231 cells to liver, bone and lung. **(a)** Confocal laser microscopy of chambers and adjacent channels at 2, 24 and 72 h after CXCR4 OE MDA-MB-231 perfusion; **(b)** Number of CXCR4 OE MDA-MB-231 cells localizing into liver, bone, lung and blank chambers; **(c)** Maximum localization extent of CXCR4 OE MDA-MB-231 cells localizing into liver, bone, lung and blank chambers; **(d)** Confocal laser microscopy of chambers and adjacent channels at 2, 24 and 72 h after claudin-2 OE MDA-MB- 231 perfusion; **(e)** Number of claudin-2 OE MDA-MB-231 cells localizing into liver, bone, lung and blank chambers; **(f)** Maximum localization extent of claudin-2 OE MDA-MB-231 cells localizing into liver, bone, lung and blank chambers; **(g)** Confocal laser microscopy of chambers and adjacent channels at 2, 24 and 72 h after Linc-ZNF469–3 OE MDA-MB-231 perfusion; **(h)** Number of Linc-ZNF469–3 OE MDA-MB-231 cells localizing into liver, bone, lung and blank chambers; **(i)** Maximum localization extent of Linc-ZNF469–3 OE MDA-MB-231 cells localizing into liver, bone, lung and blank chambers. Scale bar: 50 μm. Migration numbers and extents were counted for breast cancer cells entering chambers in individual secondary channels (n=6). In the bar charts for migration numbers and extents, statistical values on individual bars indicate comparisons between breast cancer cell migration numbers or extents into organs and those in blank groups at the same time point. Statistical values with lines indicate intergroup comparisons of breast cancer cell migration numbers or extents into organs at the same time point. *p<0.05, **p<0.01.

As shown in **Figures 4a–c**, 24h and 72h after perfusion of CXCR4 OE MDA-MB-231 cells, these cells invaded into liver, bone and lung. Compared to liver and lung, more CXCR4 OE 231 cells invaded into bone, with a greater maximum localization extent (p<0.05). **Figures 4d–f** shows that 24h and 72h after perfusion of claudin-2 OE MDA-MB-231 cells, these cells invaded into liver, bone and lung. More claudin-2 OE 231 cells invaded into liver than into bone and lung (p<0.05). At 24h, the maximum localization extent in liver was longer than in bone and lung (p<0.01). At 72h, it was longer than in lung (p<0.01), but not significantly different from bone. **Figures 4g–i** shows that 24h and 72h after perfusion of Linc-ZNF469–3 OE 231 cells, these cells invaded into liver, bone and lung. Compared to liver and bone, more Linc-ZNF469–3 OE 231 cells invaded into lung, with a greater maximum localization extent (p<0.05).

Next, we compared the differences in invasion numbers and maximum localization extent of different overexpressed 231 cells in the same organ. As shown in **Supplementary Figure S4**, at 24h post-perfusion, claudin-2 OE 231 cells had more liver invasions than Linc-ZNF469–3 OE 231 cells (p<0.01). At 72h, claudin-2 OE 231 cells had more liver invasions than the other two overexpressed 231 cells and unmodified MDA-MB-231 cells (p<0.05). For localization extent, at 24h, claudin-2 OE 231 cells had a greater maximum localization extent in liver than the other two overexpressed 231 cells and unmodified 231 cells (p<0.01). At 72h, it was greater than the other two overexpressed 231 cells (p<0.05). At 24h and 72h, CXCR4 OE 231 cells had more bone invasions than the other two overexpressed 231 cells and unmodified 231 cells (p < 0.05). For localization extent, at 24h, there was no significant difference in maximum localization extent among the cells. At 72h, CXCR4 OE 231 cells had a greater maximum localization extent in bone than the other two overexpressed 231 cells (p<0.05). At 24h, Linc-ZNF469–3 OE 231 cells had more lung invasions than the other two overexpressed 231 cells (p<0.05). At 72h, Linc-ZNF469–3 OE 231 cells had more lung invasions than claudin-2 OE 231 cells (p<0.05). For maximum localization extent, at 24h and 72h post-perfusion, Linc-ZNF469–3 OE 231 cells had a greater maximum localization extent in lung than the other two overexpressed 231 cells (p < 0.05).

### 3.4 Organotropism of PDOs cells to liver, bone and lung

**Figure 5:**
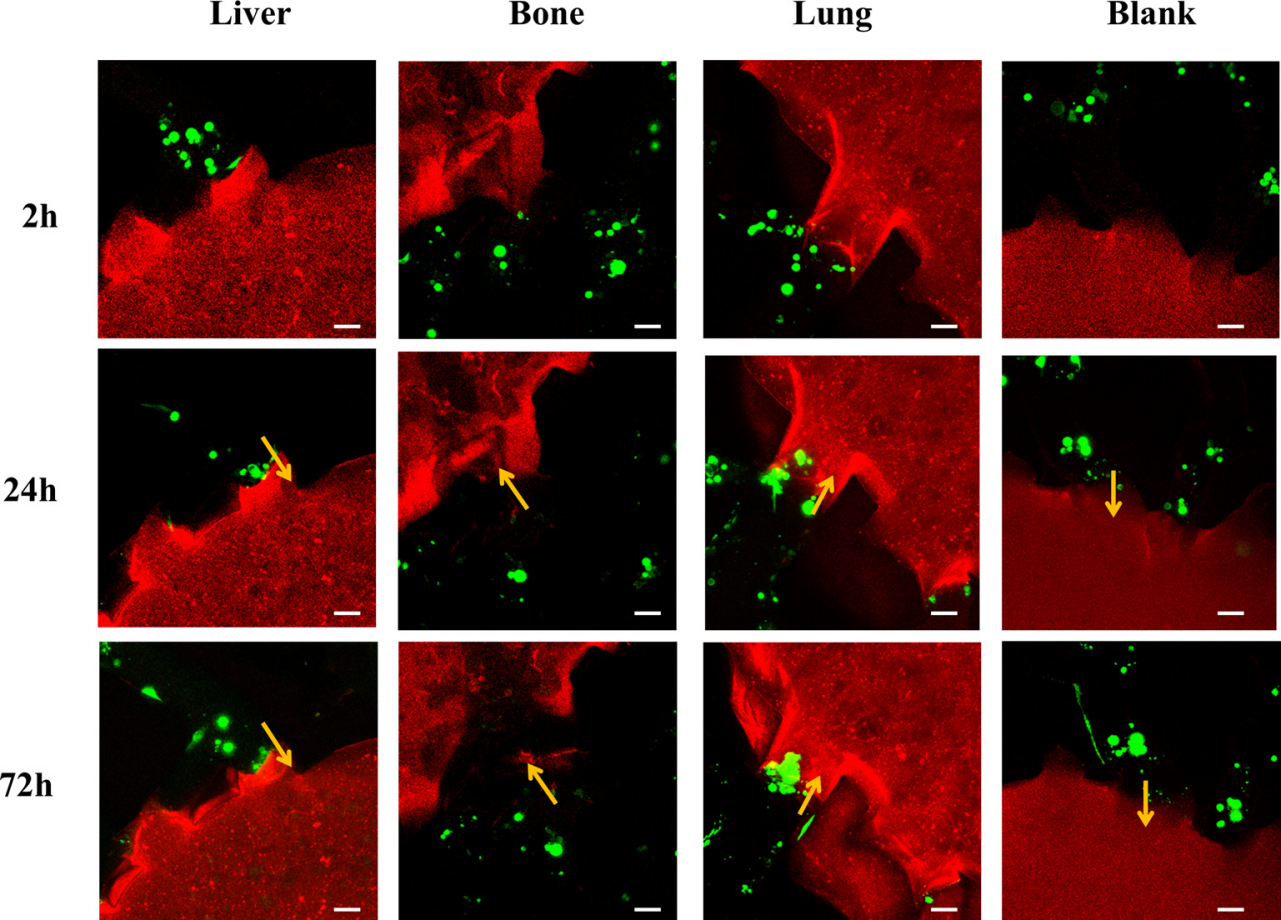
Laser confocal microscopy images of breast cancer organoid cells derived from a breast cancer patient with pulmonary metastasis perfused into the multi-organ chip at 2 hours, 24 hours, and 72 hours, showing the cells within the chamber and adjacent microchannels. Scale bar: 50 µm.

We selected a surgical specimen from a patient with advanced breast cancer presenting with pulmonary metastasis at initial diagnosis and cultured it into PDOs to evaluate the metastatic specificity of PDOs on a multi-organ microfluidic model. As shown in **Figure 5**, the PDOs cells did not invade the liver, bone, or blank pores at 2 hours, 24 hours, or 72 hours post-perfusion. However, they invaded the lung organ at 24 and 72 hours post-perfusion, albeit with a low number of invading cells and short localization extents. Given the slow growth rate of breast cancer organoids, excessive passaging may affect their biological characteristics. Therefore, only one replicate experiment was performed for the PDOs derived from a single breast cancer patient, precluding the calculation of statistical differences.

## 4 Discussion

In this study, we developed a leaf-vein microfluidic multi-organ chip to explore the organotropism of circulating breast cancer cells in common metastatic organs (liver, bone, lung), aiming to provide an early-warning platform for high-risk organ localization in breast cancer. Inspired by Murray’s law, which governs fluid transport in both plant leaf venation and mammalian vascular systems, leaf-vein chip design aims to replicate the hierarchical and reticulate structures found in real vascular systems. Our cooperative team’s previous studies revealed that leaf-vein microfluidic chips possess highly biomimetic fluidic properties, with a fluid environment closely mimicking the *in-vivo* vascular system (9–13). These properties allow for more efficient fluid distribution and nutrient exchange, which is critical for creating realistic microenvironments for studying cell behavior, offering more realistic physiological conditions for CTCs migration and colonization. Consequently, the chip design in this study drew inspiration from the biomimetic characteristics of leaf veins and further expanded its scope of application. Human normal liver, bone, and lung cells were subjected to three-dimensional culture within fibrin gels to reconstruct organ-specific tumor microenvironments, thereby mimicking the selective localization of breast cancer cells to multiple organs. S. Bersini et al. three-dimensionally cultured human bone marrow mesenchymal stem cells in collagen and confirmed that these cells produced calcium and secreted bone-marker proteins like osteocalcin, indicating the reconstruction of a bone-specific tumor microenvironment and successful simulation of breast cancer cell metastasis to the bone microenvironment (8). This study proves the feasibility of constructing organ-specific microenvironments through 3D cell culture. In our research, normal bone cells were also bone marrow mesenchymal stem cells, and we simultaneously three-dimensionally cultured liver and lung organs within the leaf-vein microfluidic chip, comprehensively presenting the selective localization characteristics of breast cancer cells in the presence of multiple organs. Previous studies have constructed multi-organ microfluidic chips. For instance, Zhiyun Xu et al. established a lung cancer model upstream in a microfluidic chip, with astrocytes, bone cells, and liver cells downstream to simulate brain, bone, and liver metastasis. They verified that lung cancer cells damage normal cells via cytokines secreted by astrocytes, bone cells, and liver cells but did not observe macroscopic tumor cell metastasis (17). Besides, many past studies on tumor cell metastasis in microfluidic chips focused on biochemical signal-induced migration, such as the chemotactic migration of tumor cells induced by epidermal growth factor (18) and CXCL12 chemokine (19). Compared to these studies, the extracellular matrix and proteins secreted by liver, bone, and lung cells in our study better mimic the complex cellular signals in the *in-vivo* environment, forming natural gradients rather than imposed nutrient or chemokine gradients.

Breast cancer subtypes show significant heterogeneity in metastatic tendencies, with different common metastatic organs. Epidemiological studies indicate that Luminal A, Luminal B, and HER-2 positive breast cancers are more prone to bone metastasis, with incidence rates of 18.7%, 30.4%, and 30.9%, respectively. HER-2 positive breast cancer also more commonly metastasizes to the liver, with a probability of 23.3%, compared to 7.9%, 13.8%, and 10.7% for Luminal A, Luminal B, and TNBC, respectively (20). TNBC patients have a higher incidence of lung metastasis than other subtypes, with a rate of about 31%, higher than Luminal A (17%), Luminal B (14%), and HER-2 positive breast cancer (25%) (21). In our study, after perfusing three breast cancer subtypes for 24h and 72h, the number of invasive breast cancer cells and the maximum localization extent in the liver, bone, and lung were greater than in blank chambers. MCF-7 cells showed the most invasion and greatest localization extent in bone; SKBR3 cells had more invasion and greater localization extent in liver and lung; MDA-MB-231 cells showed no difference in invasion number among liver, bone, and lung. The number of invasive MDA-MB-231 cells in liver, bone, and lung was higher than that of MCF-7 cells, and the number of invasive MDA-MB-231 cells in bone was higher than that of SKBR3 cells. There was no difference in the maximum localization extent of the three subtypes in the liver; MDA-MB-231 and MCF-7 cells had a greater localization extent than SKBR3 cells in bone; MDA-MB-231 and SKBR3 cells had a greater localization extent than MCF-7 cells in lung. Compared to breast cancer metastasis epidemiology, hormone-receptor positive MCF-7 cells preferentially localize in bone, HER-2 positive SKBR3 cells to liver and lung, which aligns with epidemiological results. However, SKBR3’s low bone localization tendency contradicts epidemiological data. TNBC MDA-MB-231 cells showed no preference in localization to liver, bone, or lung. Our results partially match the actual metastasis trends of breast cancer, and discrepancies may arise from tumor heterogeneity and changes in the genetic background and biological behavior of long-cultured breast cancer cell lines.

To verify the effectiveness of our leaf-vein microfluidic multi-organ tropism model, based on reported molecular mechanisms regulating breast cancer metastasis, we constructed three overexpressed MDA-MB-231 cell lines: CXCR4 OE 231, claudin-2 OE 231, and Linc-ZNF469–3 OE 231. Results showed that CXCR4 OE 231, claudin-2 OE 231, and Linc-ZNF469–3 OE 231 cells invaded bone, liver, and lung organs respectively in the highest numbers, with statistical significance compared to other cells. However, the maximum localization extents of these modified cell lines in the bone, liver, and lung were the greatest, no statistically significant differences were found compared with unmodified cells. This indicates gene modification can increase the number of MDA-MB-231 cells localizing in specific organs, consistent with previous studies (14–16). Tumor organoids can predict clinical treatment responses in precision medicine (22). To further validate the effectiveness of the organotropism model, this study integrated tumor organoids with microfluidic chips and assessed the metastatic trend of PDOs from a patient with advanced pulmonary metastasis within this model. The results demonstrated that the PDOs selectively invaded the lung organ, which is consistent with the patient’s actual metastatic pattern. This finding indicates that the leaf-vein-inspired microfluidic multi-organ tropism model constructed in this study is capable of predicting patient-specific organotropism behavior.

In this study, fibrin gel was chosen as the extracellular matrix. Beyond fibrin gel, collagen I and Matrigel are also widely used as 3D culture matrices. Fibrin gel exhibits remarkable biocompatibility and biodegradability, being a natural biomaterial that the body can gradually metabolize without triggering significant immune or toxic responses (23). Additionally, it facilitates various cell behaviors, such as proliferation, migration, and differentiation, which are essential for the construction of functional organ models (24). The mechanical properties of fibrin gel, including its elasticity, can be readily adjusted by altering the concentrations of fibrinogen and thrombin or by modifying the polymerization conditions, allowing it to mimic the mechanical microenvironments of diverse tissues (23). Collagen I is widely used in tissue engineering and tumor studies due to its excellent cell adhesion and migration support capabilities, as well as its biocompatibility, mechanical strength, degradability, and limited immunogenicity (25). However, its relatively stiff mechanical properties may not be suitable for studies requiring softer tissue simulation (26). Matrigel is also extensively used in cancer research for its ability to support cell heterogeneity and spontaneous cell organization. Yet, its complex composition and animal-derived components can influence experimental reproducibility and controllability (27). In comparison, fibrin gel provides a more comprehensive solution for our research needs. Our collaboration with Prof. He’s team further reinforced our choice of fibrin gel. Their research demonstrated that fibrin gel can effectively create a biomimetic vascular system integrated with organ-specific chambers in the leaf-vein microfluidic chip (12). Building on their work and considering the advantages of fibrin gel over other matrices, we also utilized fibrin gel in the leaf-vein microfluidic chip for our study.

Main limitations of this study are as follows: (i) The constructed liver, bone, and lung organs lack verification of physiological functions via specific protein secretion (e.g., osteocalcin, pulmonary surfactant), potentially affecting the authenticity of metastasis microenvironment simulation; (ii) Analysis of breast cancer cell organ-tropism is based on only six microfluidic chambers, requiring a larger sample size for more reliable conclusions; (iii) Only one PDO sample from a late-stage patient was included, due to the low surgery rate of untreated advanced-stage patients and consent requirements for tissue acquisition, which significantly restricted our access to multiple patient samples. As a result, our findings may not fully capture the extensive tumor heterogeneity typically present across different patients. (iv) The current model doesn’t incorporate key physical factors like endothelial barrier permeability and blood flow shear stress gradients, possibly reducing the biological authenticity of CTC extravasation. (v) The absence of non-target organ controls and the potential impact this may have on the interpretation of our results.

In future studies, we plan to conduct *in vivo* xenograft experiments, such as intracardiac or tail vein injection into mice, to validate whether the cells that localize in our microfluidic chip also metastasize to similar organs *in vivo*. Additionally, we aim to incorporate immune cells, stromal fibroblasts, and extracellular matrix remodeling into our model to better simulate the *in vivo* microenvironment. We also plan to add endothelial cells (e.g., HUVECs) to line the channels of our chip to better mimic the vascular interface. These enhancements will significantly improve the model’s ability to simulate the early stages of metastasis and provide a more comprehensive understanding of the metastatic process.

## 5 Conclusion

This study successfully developed a multi-organ organotropism model for circulating breast cancer cells based on a leaf-vein microfluidic system, offering an innovative platform to explore their organ-specific adhesion tendencies in common organs like liver, bone, and lung. By perfusing and analyzing three breast cancer subtypes in this model, we found significant differences in invaded number and extent of organ-specific localization among subtypes in different organs, partially matching breast cancer metastasis epidemiology. Further gene-modified MDA-MB-231 cells showed increased invasion and greater localization extent in corresponding organs, confirming gene modification’s targeted regulation of organotropism and the model’s precision and reliability. Additionally, the experiments integrating tumor organoids with microfluidic chips further demonstrated the potential application value of this model in predicting patient-specific organotropism patterns. It provides a powerful tool for early warning of high-risk organ localization in breast cancer. This achievement offers crucial theoretical and technical support for precision treatment and individualized management of breast cancer in clinical practice, promoting a more precise and efficient direction for its diagnosis and treatment.

## Data Availability

The original contributions presented in the study are included in the article/**Supplementary Material**. Further inquiries can be directed to the corresponding authors.
