# Supplementary material for: Leaf-vein-inspired multi-organ microfluidic chip for modeling breast cancer CTC organotropism
Source: Front Oncol. 2025 May 29;15:1602225. doi: 10.3389/fonc.2025.1602225 (PMC12158725; doi:10.3389/fonc.2025.1602225)
Supplement: Supplementary file 1 [file DataSheet1.docx]

**Leaf-Vein-Inspired Multi-Organ Microfluidic Chip for Modeling Breast Cancer CTC Organotropism**

**Supplementary materials**


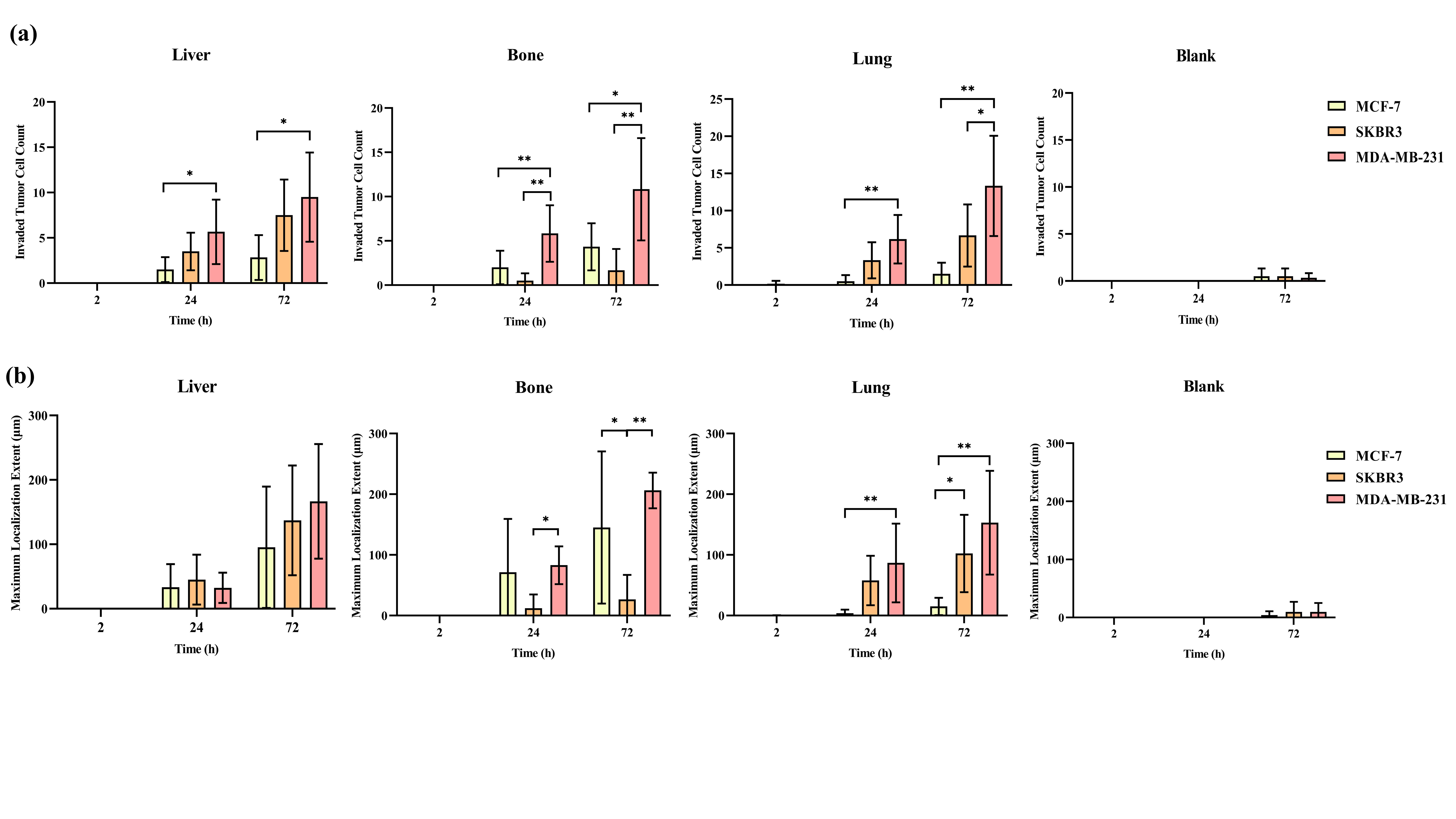


**Figure S1.** Comparison of the invaded tumor numbers and the maximum localization extent of different breast cancer cell subtypes transferred to liver, bone, lung, and blank chambers. (a) Comparison of invaded tumor numbers; (b) Comparison of the maximum localization extent. ^*^*p*<0.05, ^**^*p*<0.01.


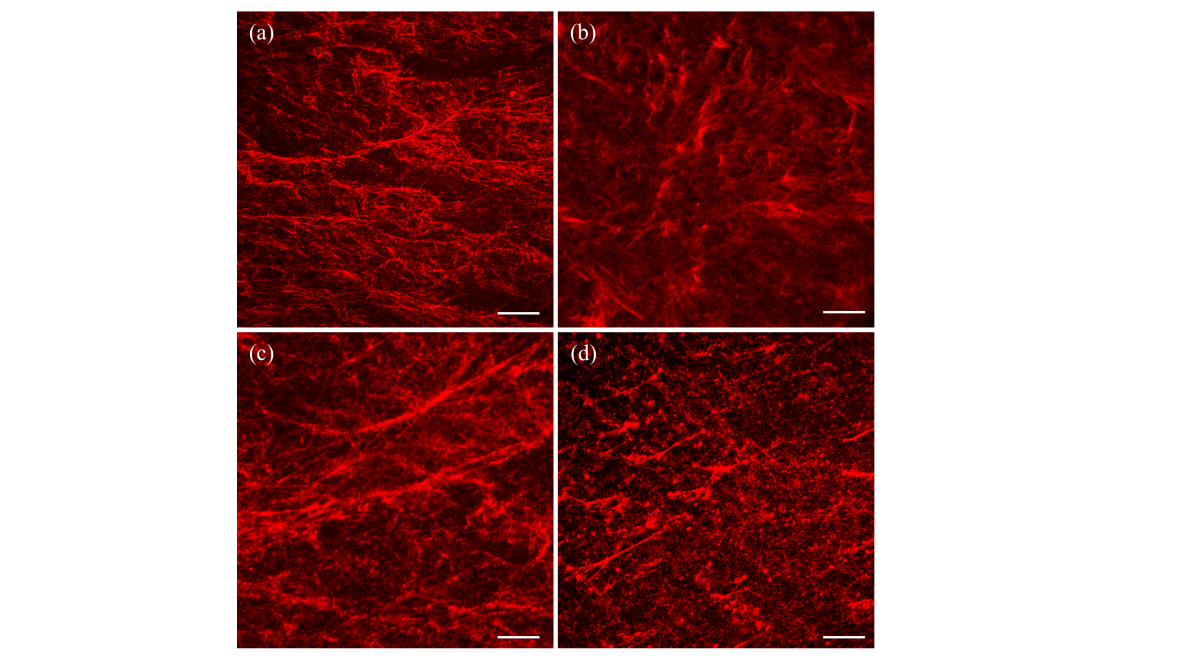


**Figure S2.** Confocal laser microscopy images of the gel structures in different groups. (a) Fibrin gel structure seeded with LO2 cells; (b) Fibrin gel structure seeded with h-MSC cells; (c) Fibrin gel structure seeded with HFL-1 cells; (d) Fibrin gel structure without any cell seeding. Scale bar: 10μm.


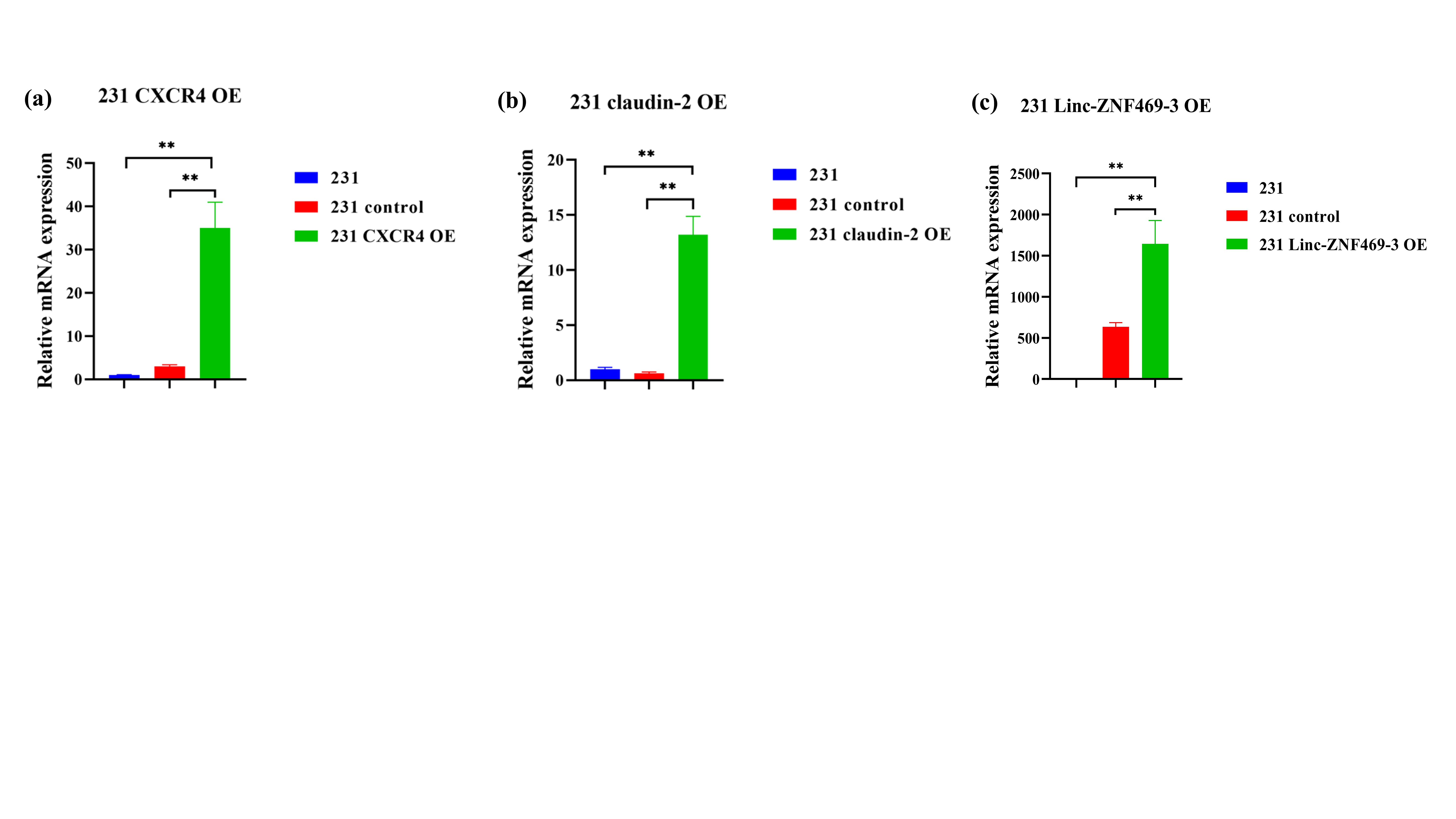


**Figure S3.** Validation of the three overexpressed genes in MDA-MB-231 constructs by q-PCR. (a) Relative mRNA expression levels of CXCR4-overexpressing cells and the control group; (b) Relative mRNA expression levels of claudin-2-overexpressing cells and the control group; (c) Relative mRNA expression levels of Linc-ZNF469-3-overexpressing cells and the control group. ^**^*p*<0.01.


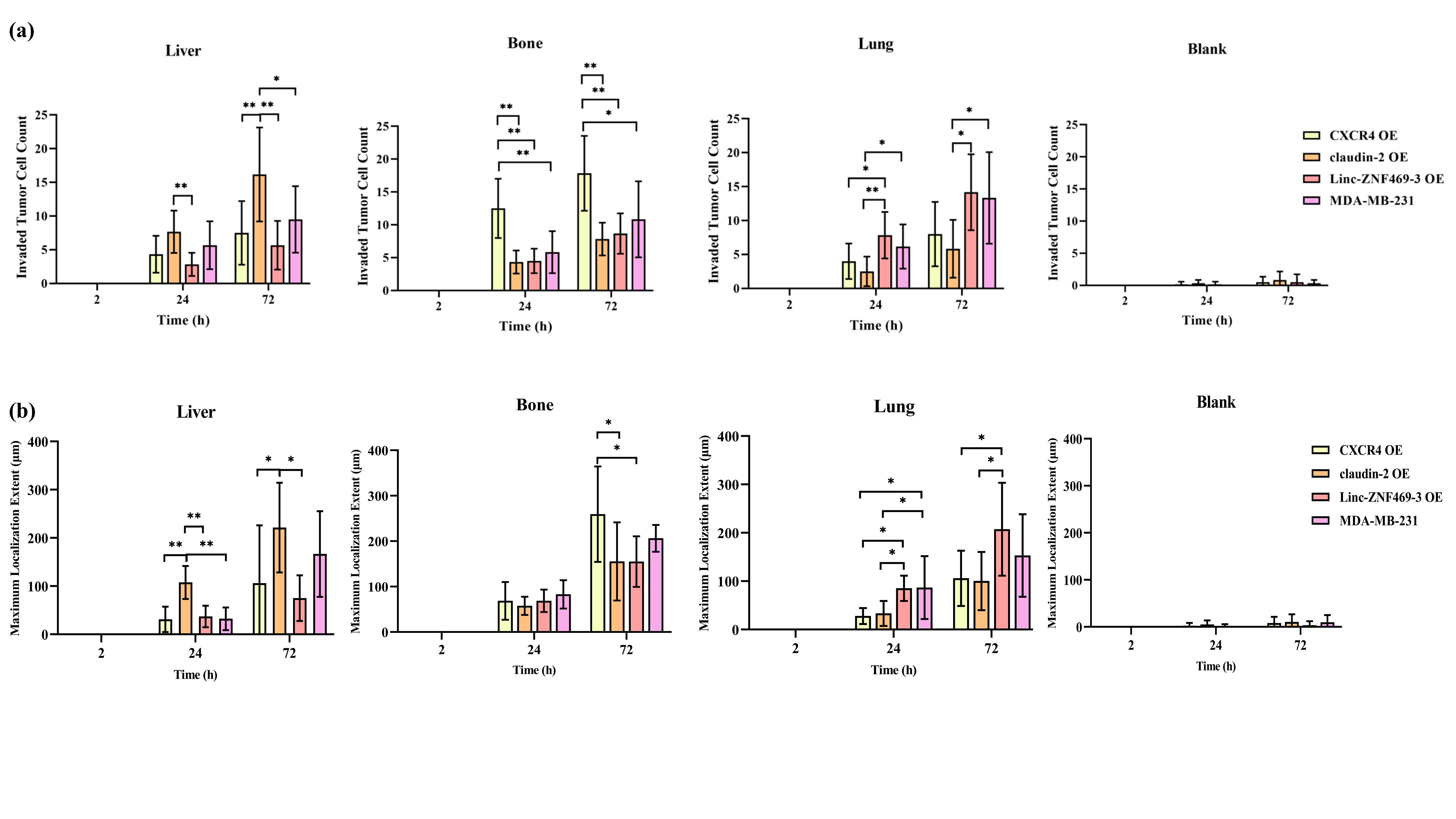


**Figure S4.** Comparison of invaded numbers and maximum localization extent of differently overexpressed MDA-MB-231 cells transferred to liver, bone, lung, and blank chambers. (a) Comparison of migration numbers; (b) Comparison of maximum localization extent. ^*^*p*<0.05, ^**^*p*<0.01.
